# Factors Associated with Dietary Restriction and Emotional and Uncontrolled Eating in Adults from Spanish-Speaking Countries during the COVID-19 Confinement: Results of the CoV-Eat Project

**DOI:** 10.3390/nu14224866

**Published:** 2022-11-17

**Authors:** Anna Vila-Marti, Catalina Ramírez-Contreras, Evelia Apolinar-Jiménez, Pía Rojas-Cárdenas, Desirée Valera-Gran, Rafael Almendra-Pegueros, Eva María Navarrete-Muñoz

**Affiliations:** 1 Research Group M3O, Methodology, Methods, Models and Outcomes of Health and Social Sciences, Facultat de Ciències de la Salut i el Benestar, Universitat de Vic—Universitat Central de Catalunya, 08500 Vic, Catalonia, Spain; 2 Departamento de Nutrición, Ciencias de la Alimentación y Gastronomía, Facultad de Farmacia y Ciencias de la Alimentación, Universidad de Barcelona, 08028 Barcelona, Spain; 3 Instituto de Investigación en Nutrición y Seguridad Alimentaria (INSA-UB), Universidad de Barcelona, 08921 Santa Coloma de Gramenet, Spain; 4 Unidad de Metabolismo y Nutrición, Departamento de Investigación, Hospital Regional de Alta Especialidad del Bajío, León 37660, Mexico; 5 Carrera de Nutrición y Dietética, Facultad de Ciencias de la Salud, Universidad Adventista de Chile, Chillán 3780000, Chile; 6 Escuela de Nutrición y Dietética, Universidad del Bío-Bío, Chillán 3800708, Chile; 7 Grupo de Investigación en Terapia Ocupacional (InTeO), Universidad Miguel Hernández, 03202 Elche, Spain; 8 Institut d’Investigació Biomèdica Sant Pau (IIB SANT PAU), Sant Quintí 77-79, 08041 Barcelona, Spain; 9 Red de Nutrición Basada en la Evidencia, Academia Española de Nutrición y Dietética, 31006 Pamplona, Spain; 10 Instituto de Investigación Sanitaria y Biomédica de Alicante (ISABIAL), 03010 Alicante, Spain

**Keywords:** COVID-19, confinement, feeding behaviour, three-factor eating questionnaire

## Abstract

The first COVID-19 confinement has led to changes in the population’s behaviour. However, little has been analysed about the changes in eating behaviour beyond the decrease in adherence to healthy dietary patterns. The aim of the CoV-Eat project was to identify factors related to each of the following eating behaviours (EB): cognitive restraints (CR), uncontrolled eating (UE), and emotional eating (EE) in adults from Spanish-speaking countries. Participants completed an anonymous online survey. EB was assessed using the Three-Factor Eating Questionnaire and the scores were classified into tertiles. Socio-demographic characteristics and lifestyle habits including physical activity, sleep quality, screen use, smoking consumption, and relationship with food were also collected. A total of 9849 participants from 21 countries were included in this study. The median of CR, UE, and EE behaviours was 15, 24, and 9, respectively. We observed that higher age, days of confinement, number of times leaving home in the last week, changes in sleep quality, and their relationship with food were negatively associated with CR, EE, and UE, while being a man was positively associated with an increased in all these EBs. A higher household income was negatively associated with a higher score of CR, and a higher education level (>primary) was positively associated with a medium score of EE. Lower physical activity was a factor negatively associated with a medium score of EE and UE. Higher screen use showed the same negative association for a medium score of EE and UE and a high score of CR. In addition, higher tobacco consumption was found to be a protective factor against having a medium or high score of CR and EE. In conclusion, some sociodemographic characteristics and lifestyle changes may be important factors for EB and should be considered in emergency situations such as confinement to prevent risky eating behaviour.

## 1. Introduction

Nothing foreshadowed at the beginning of 2020 that the world would come to a standstill due to a highly contagious virus such as SARS-CoV-2. However, in a few days and from different countries, we saw how the World Health Organization classified this as a pandemic infection (11 March 2020) and how various governments had to take drastic measures to guarantee the population’s safety. Home confinement was established on a mandatory basis in many countries and voluntarily in others, leading, indubitably, to an unforeseen situation that has caused enormous stress and, probably, changes in habits [1,2].

The interest in exploring the effects of this social isolation period on health has generated ad hoc research focused on lifestyle choices or changes during this lapse of time. Several studies conducted during the COVID-19 confinement showed changes in eating and sleeping schedules, a reduction in energy expenditure and physical activity, an increase in unhealthy habits such as smoking, alcohol, and other drugs, and higher screen time, but also beneficial practices such as an increase in time dedicated to cooking healthier homemade recipes [3,4]. Moreover, the ECLB COVID-19 International Online Survey, a study conducted in several countries in Europe, America, Asia, and North Africa with 5000 participants showed that home confinement led to significant psychological changes that negatively impacted food intake, increasing the consumption of unhealthy and ultra-processed foods, snacks between meals, the number of main meals, eating out of control, and alcohol consumption [1,5,6]. This study also observed other lifestyle changes, including reduced physical activity, increased sitting time, and less sleep quality compared to the pre-pandemic situation [1,5,6]. In the young population, a study conducted on 1820 individuals from Southern California showed that a large majority of participants (69%) increased food intake as a way of coping with the pandemic, resulting in weight gain [7]. In addition, one study conducted with 1084 participants from Mexico during the lockdown reported that women had a healthier diet than men, consisting mainly of a high intake of fresh food and a reduction in sweets and desserts [8]. However, some studies observed that women who followed a diet rich in unhealthy foods (i.e., “eating high fat or sugary foods”) and overate (i.e., “eating more food than usual”) during the first COVID-19 lockdown experienced increased depressive symptoms [7,9]. On the contrary, a European survey with 36,185 adults found an improvement in diet quality during social isolation due to greater participation in home cooking [4].

Despite the rapidly growing literature on food practices or nutrition during the first COVID-19 confinement, few studies have explored eating behaviours such as dietary restriction, uncontrolled eating, and/or emotional eating. Among these eating behaviours, emotional eating has been the most studied. Two studies conducted on the Spanish population observed that the prevalence of emotional eaters during this home-staying period ranged from 10% [10] to 21.8% [2], while the proportion of those categorised as very emotional eaters was 11% [2]. However, a survey conducted on Turkish individuals showed that 75.7% of the participants were emotional eaters at different levels, ranging from low (36.9%) to very emotional eaters (4.0%) [11]. Moreover, some studies have explored the factors associated with eating behaviours during this first confinement period. In the Italian population, a study observed that emotional eating was significantly associated with a higher level of negative emotions such as anxiety [12]. A study on psychological distress during the COVID-19 pandemic disclosed that eating behaviours, namely, emotional eating and uncontrolled eating, were associated with personality dimensions such as perfectionism [13]. Finally, a study with data from 724 Brazilian individuals showed that emotional eating, binge eating, physical inactivity, and vespertine chronotype were negatively correlated with a high score in emotional eating, binge eating, physical inactivity, and vespertine chronotype during the COVID-19 isolation period [14].

In light of available evidence, the findings from the association of eating behaviours with other potential influencing factors during the first COVID-19 confinement are still at an emerging stage. To date, many questions remain on eating behaviours during this isolation period and their effects on health. Therefore, the present study aimed to (1) describe eating behaviours, including dietary restriction, emotional eating, and uncontrolled eating, and (2) explore the factors associated with these eating behaviours among adults during the first confinement due to the COVID-19 pandemic in Spanish-speaking countries.

## 2. Materials and Methods

### 2.1. Study Design and Participants

The CoV-Eat project is a cross-sectional online survey aimed at exploring eating behaviours in people from Spanish-speaking countries. Detailed information on the study protocol has been published previously [15]. The data of the study participants were collected between April and June 2020 using a Google Forms platform according to the criteria proposed by the Checklist for Reporting Results of Internet E-Surveys (CHERRIES) [16]. To avoid duplicate responses, we applied the option in Google Forms “limit to one response”. The target population was adults between 18- and 65-year-old citizens from 21 participating countries: Andorra, Argentina, Bolivia, Chile, Colombia, Costa Rica, Cuba, Ecuador, El Salvador, Spain, Guatemala, Honduras, México, Nicaragua, Panamá, Paraguay, Perú, Puerto Rico, Dominican Republic, Uruguay, and Venezuela. A criterion for participating in the study was that the participants should be compulsorily or voluntarily confined at home for at least seven days in a row. Potential participants were recruited through a dissemination campaign via social networks using snowball samplings such as Twitter, LinkedIn, Facebook, Instagram, Telegram, and WhatsApp. This sampling method is particularly suitable during a confinement situation that significantly reduces the population’s mobility and social contact. Thus, the online distribution of the survey enabled large numbers of people to access it quickly. Of the 10,281 participants who started the survey, 296 were excluded from the analysis due to missing data for the outcome and exposure variables. Finally, we included a sample of 9849 (95.8%).

The survey consisted of questions organised into the following sections: 1. Questionnaire of eating behaviour (Three-Factor Eating Questionnaire-R18, TFEQ-R18-SP), 2. Sociodemographic and health information (anthropometrics, chronic illnesses, and sociodemographic data; and 3. Lifestyle (social isolation, lifestyle, and acquisition of foods). Sections 2 and 3 included closed- and open-ended ad hoc questions. The survey took approximately 10–15 min. Detailed information of each section is described as follows:

(1) To assess eating behaviour, we used the validated Spanish version of the Three-Factor Eating Questionnaire-R18 (TFEQ-R18-SP) [17]. This test measures three different types of eating behaviour: (a) cognitive restraint (CR), defined as the conscious restriction of the consumption of foods aimed at controlling the corporal weight and/or promoting the loss of weight; (b) emotional eating (EE), defined as eating as a way to suppress negative emotions; and (c) uncontrolled eating (UE), referred to the tendency to eat more than the usual because of a loss of control on the consumption with a subjective feeling of hunger. The questionnaire consists of 18 items divided into three subscales: CR (six items), EE (three items), and UE (nine items). The items can be scored on a four-point scale (definitively true: 1, mainly true: 2, mainly false: 3, definitively false: 4). The total score of each subscale can be obtained from the sum of the respective items. Higher scores in the subscales indicated greater CR, EE, and UE.
(2) In the section on sociodemographic and health information, we collected the following data: (a) self-reported basic anthropometrics data: weight (kg), height (cm), abdominal circumference (cm); (b) health status information: previous diagnosis of chronic illnesses: diabetes, hypertension, cancer, obesity, and time passed from the diagnosis; and (c) sociodemographic data: country of residence, sex (woman/man), age, educational level (primary, secondary, university), employment situation prior to the pandemic and current (works, teleworking, retired, stopped/earning subsidy, stopped/without earning, student, unpaid domestic work, or other situations), marital status (single, coupled up), and number of people living at home.
(3) The last section included information about lifestyles: (a) days of confinement, type of cohabitation (alone, in a family without children, in a family with children), frequency and reason for leaving the house; (b) data related with lifestyles before and during home confinement: sleep quality, physical activity, screen use, tobacco consumption, relationship with food; and (c) data related with the purchase of foods: frequency and monetary cost in the purchase of foods in the last week and frequency of food delivery to home.

Previously to collect the data, this study’s protocol was published in the Open Science Framework (https://osf.io/dz9s7/) to allow a pre-evaluation by experts in the area and bioethics. Furthermore, this study complied with the premises established in the Declaration of Helsinki for research on human beings and according to the Guide for Health Research during Disasters, Emergencies, or Epidemics outbreaks [18]. The nature of this study guarantees minimal risk and respects the granting of informed consent by each research person.

The researchers are committed to protecting the access to the data obtained from the questionnaire through electronic devices and the Internet to safeguard each participant’s data, and no sensitive information that can identify them was collected. In addition, informed consent for participation was obtained online following the principle of autonomy.

### 2.2. Study Variables

#### 2.2.1. Eating Behaviours

Total scores for each subscale (CR from 6 to 24, EE from 3 to 12, and UE from 9 to 35 points) were calculated. Then, the participants were classified according to the tertiles of CR (<15, 15–16, >16 points), EE (<9, 9–10, >10), and UE (<14, 24–26, >26 points).

#### 2.2.2. Covariates

The sociodemographic characteristics considered for the analysis were: age in years; sex (women and men); marital status (single, coupled up); education level (≤primary; >primary); household income (lower, medium, higher); the number of people living at home (0–2, >2); days of confinement in tertiles (7–21, 22–31, and >31 days); and the number of times they left their homes during the last week (0, 1 and, >1). In addition, we included the following lifestyle variables: sleep quality (as before the COVID-19 confinement, lower, and higher); physical activity (as before the COVID-19 confinement, lower, and higher), screen use (as before the COVID-19 confinement, lower, and higher), tobacco consumption (no smoker, as before the COVID-19 confinement, lower, higher, and ex-smoker), and relationship with food (as before the COVID-19 confinement, different).

### 2.3. Statistical Analyses

Statistical analyses were conducted using software R, version 4.0.2 (R Foundation for Statistical Computing, Vienna, Austria; http://www.r-project.org/). We applied bilateral statistical tests with a significance level set at 0.05. In addition, the normal distribution of the continuous variables was checked by the Lilliefors correction Kolmogorov–Smirnov test.

The CR, EE, and UE were described using a median and interquartile range (IR). The sociodemographic and lifestyle characteristics were presented as percentages in categorical variables and median and IR in continuous variables. Moreover, the sociodemographic and lifestyle characteristics according to the tertiles of CR, EE, and UE were also described using percentages.

Predictors of the medium and higher scores of eating behaviour variables (i.e., CR, EE, and UE) were assessed by multiple Poisson regression models with robust variance based on the sandwich estimation of Huber to obtain prevalence ratios (PR) and their 95% confidence interval (CI) [19,20]. Because of non-convergence, Poisson regression with robust variance was applied instead of the log-binomial regression [21].

## 3. Results

In the present study, the median (interquartile range) of CR, UE, and EE in the participants of CoV-Eat was 15 (14; 17), 9 (7; 11), and 24 (21; 28), respectively. The sociodemographic and lifestyle characteristics are shown in Table 1. The median age was 31 years, and 80.1% of the participants were women. Most participants were coupled up, had medium income, and had secondary or university studies. The median of days of confinement was 23, and 51.3% of them left their homes during the last week more than one time. According to lifestyle characteristics, most participants reported changes in their sleep quality, physical activity, screen time, and food relationship routines due to the COVID-19 confinement. In addition, 84.9% of participants reported being non-smokers or ex-smokers.

Table 2 shows associated factors with medium and high scores of CR in the participants from the CoV-EAT project. Statistical significant predictors of the medium score of CR (15–16 points) were being older (PR_31–40_ vs. _18–30_ = 0.92; PR_41–65_ vs. _18–30_ = 0.90), being men (PR = 1.14), leaving home more than once during the last week (PR_>1_ vs. _0_ = 0.93), changes in sleep quality (PR_higher_ vs. _as before_ = 0.92; PR_lower_ vs. _as before_ = 0.94), and relationship with food (PR_different_ vs. _as before_ = 0.89). In addition, we observed that being older (PR_31–40_ vs. _18–30_ = 0.91; PR_41–65_ vs. _18–30_ = 0.87), being men (PR = 1.30), household income (PR_medium_ vs. _lower_ = 0.90; PR_higher_ vs. _lower_ = 0.80), days of confinement (PR_22–31 days_ vs. _< 22 days_ = 0.92; PR_>31days_ vs. _<22days_ = 0.90), the number of times they left the house during the last week (PR_1_ vs. _0_ = 0.91; PR_>1_ vs. _0_ = 0.87), changes in sleep quality (PR_higher_ vs. _as before_ = 0.88; RP_lower_ vs. _as before_ = 0.87), screen time (PR_higher_ vs. _as before_ = 0.91), tobacco consumption (PR_higher_ vs. _never smoker_ = 0.81; PR_lower_ vs. _never smoker_ = 0.87; PR_ex-smoker_ vs. _never smoker_ = 0.83), and relationship with food (PR_different_ vs. _as before_ = 0.69) were significantly associated with the high score > 16 of CR.

Table 3 shows the predictors of medium and high scores of EE in the participants in the CoV-EAT project. We observed that being older (PR_41–65_ vs. _18–30_ = 1.08), being men (PR = 1.22), and having an educational level higher than primary studies (PR = 1.09) were significantly positively associated with the medium score of EE (9–10 points), while a higher number of days of confinement (PR = 0.93), lower sleep quality (PR = 0.88), lower physical activity (PR = 0.86), higher screen use (PR = 0.85), being an ex-smoker (PR = 0.88), and changes in the relationship with food (PR = 0.67) were negatively associated with a medium score of EE. Moreover, significant statistical predictors of the high score of EE (>10 points) were older (PR_31–40_ vs. _18–30_ = 0.95; PR_41–65_ vs. _18–30_ = 0.92), men (PR = 1.11), leaving home more than once during the last week (PR_>1_ vs. _0_ = 0.92), and changes in sleep quality (PR_higher_ vs. _as before_ = 0.92; PR_>lower_ vs. _as before_ = 0.95), tobacco consumption (PR_higher_ vs. _never smoker_ = 0.87), and relationship with food (PR_different_ vs. _as before_ = 0.91).

The multivariate models for the factors associated with the medium (24–26 points) and high (>26 points) scores of UE can be observed in Table 4. The age of participants was positively associated with a medium score of UE (PR_31–40_ vs. _18–30_ = 1.08; PR_41–65_ vs. _18–30_ = 1.18) and a high score of UE (PR_41–65_ vs. _18–30_ = 0.93). In addition, men presented a higher UE score compared with women (PR = 1.11), while having secondary or university studies were positively associated with a medium score of UE (PR = 1.16). The participants with more than 31 days of confinement compared to those with less than 21 days had a negative association with medium and high scores of UE (PR = 0.87 and 0.95, respectively). Regarding the changes in lifestyles, a negative and significant association between a medium score of UE and lower sleep quality (PR = 0.90), lower physical activity (PR = 0.86), higher screen use (PR = 0.86), and relationship with food (PR = 0.73) compared to the situation before confinement was observed. In addition, we also found a negative and significant association between a higher score of UE and lower sleep quality (PR = 0.95) and the relationship with food (PR = 0.90) compared to the situation previous to confinement.

## 4. Discussion

Previous studies have described eating behaviours in the complex situation experienced during the strictest period of confinement; however, the association between eating behaviours and their potential determinants during this particular situation due to the COVID-19 pandemic has been little explored. In this study, we have observed that the factors associated with a medium and high score of CR were age, sex, the number of times leaving home during the last week, changes in sleep quality, and relationship with food. We have also found a significant association between medium and high EE and age, sex, sleep quality, tobacco consumption, and relationship with food. Moreover, the findings indicated that age, sex, days of confinement, lower sleep quality, and relationship with food were significant predictors of having a medium and high score of UE. To our knowledge, this is the first time a study has determined the factors associated with eating behaviour during COVID-19 confinement from an accurate epidemiological approach. In this respect, our findings may suppose good evidence to design interventions to prevent and reduce problematic eating behaviours.

In our study, we observed that the medians of CR, EE, and UE were 15, 9, and 24, respectively. This score was slightly higher than the results observed in previous Spanish research conducted before the COVID-19 pandemic, which reported means (standard deviation) of 11.7 (4.11), 5.1 (2.6), and 17.5 (6.2) for CR, EE, and UE, respectively [17]. Since changes in diet and maladaptive eating behaviours such as EE and UE have been linked to mental health issues [7,9,10,12,13], these results, although indirectly, might be explained by the increase in anxiety and depression symptoms during the first confinement [22,23,24]. However, we did not assess psychological distress in the study participants, thereby preventing us from supporting this assumption. Regarding the results of EE, we found that a higher number of days of confinement were negatively associated with a medium score of EE. These results may be partially consistent with the study by Cecchetto et al. who found that EE was significantly reduced in the second phase (more relaxed measures) compared to the first phase (confinement) of the pandemic [12]. In our study, we observed that changes in some lifestyle habits were predictors of the medium and high scores of EE. These results cannot be compared and contrasted with prior evidence on factors associated with EE during confinement; however, sleep quality [25] and tobacco use [26] have previously been associated with quality of life. As reported by Cechetto et al. the increased EE during the COVID-19 pandemic confinement was predicted by higher depression and anxiety, and partially by the quality of personal relationships and the quality of life [12]. Importantly, this study suggests higher tobacco consumption during confinement may have been used as a stress relief strategy, being protective against increased CR or EE during confinement. However, this result must be interpreted with caution, as we did not measure perceived stress and cannot affirm that this sample was in psychological distress. Nonetheless, previous studies have suggested that tobacco consumption was increased to cope with psychological issues during the pandemic social isolation [27,28], which raises a serious concern about unhealthy habits as a coping strategy. On the contrary, higher sleep quality was found to be a protective factor against a higher score of EE, suggesting that higher sleep quality could be served as an indicator of emotional regulation. However, the protective action of poor sleep quality against increased EE can raise further health concerns about the consequences of stressful situations such as COVID-19 confinement.

The results also showed that age was a significant factor for CR, EE, and UE during the confinement. Overall, being older was more likely to resist these eating behaviours. This finding is consistent with prior literature supporting that emotional health and regulation (i.e., ability to resist) improve with age [29]. Moreover, our results also displayed that higher household income might protect against increased CR. There is growing evidence about the link between socioeconomic inequalities and mental health problems [30], which may explain the protective effect of a medium or higher income on CR. However, although prior research on the neurobiological correlates of self-regulation and socioeconomic features might support that people with a favourable social and economic status could manage more efficiently self-regulatory behavioural processes, we did not observe this protective effect on resisting EE and UE. In fact, we found that a higher education level was a significant factor in having a medium score of EE. Although the lack of evidence on social determinants of EE during confinement prevents us from making a direct comparison, a Turkish study conducted before the COVID-19 pandemic showed that higher educational status was associated with EE [31]. However, the underlying explanation for this association remains unclear, requiring further exploration.

The evidence seems to support the relationship between eating behaviour and psychological distress. A study conducted in three Spanish-speaking countries (Chile, Colombia, and Mexico) during the COVID-19 confinement showed that the values of CR, EE, and EU were higher in people who perceived having suffered from anxiety during this time [32]. Moreover, this study indicated that women had higher scores of CR and EE, and respondents with university studies presented higher UE [32]. The potential hypothesis that the relationship between eating behaviour and psychological distress can be attributed to sex differences is consistent with the results reported by Elmacıoğlu et al. [33]. They observed that EE and UE behaviours were more prevalent in women, with no significant differences being found with the CR [33]. In line with this, a study conducted on the Portuguese population showed that the psychosocial impact of the first confinement is linked to women, younger, higher EE, and UE [34]. By contrast, our results showed that men had a higher probability of greater CR, EE, and UE compared to women. Although these results are inconsistent with previous research, and we have no apparent response to why men were more likely to present increased CR, EE, and UE, the statistical analysis of the present study supports this association. This study is the first to examine the direct association of the effect between sex and the prevalence of problematic eating behaviours during the first COVID-19 lockdown. However, more research is needed to explore in depth how social determinants, including sex, may be associated with problematic eating behaviours during stressful situations such as COVID-19 confinement.

The findings also showed that changes in lifestyles such as physical activity and screen use were factors associated with eating behaviours during the first social isolation lockdown quarantine. In line with our results, a recently published systematic review about the relationship between mental health and EE during the COVID-19 pandemic indicated that reduced physical activity, increased sedentary behaviours, and higher social media use were risk factors for the development of EE in this period [35]. In parallel, this systematic review also pointed out that EE may be a risk factor for the development of an addiction to eating, overeating, or UE. On the other hand, although we did not measure food intake, we asked the study participants if they have changed their relationship with food during confinement. As a result, we found that a change in the relationship with food (i.e., different to before) was a protective factor against CR, EE, and UE. However, the way we measured the relationship with food prevented us from elucidating what kind of changes were made in relation to food, and, therefore, we cannot provide a clear explanation for this association. In the present study, we also observed that changes in sleep quality were predictors of eating behaviours. Our results showed that higher and lower sleep quality were associated with a lower probability of increased CR, EE, and UE. Previous research has indicated that these problematic eating behaviours were associated with greater insomnia [36] and poor sleep quality [36,37]. Potential explanations for this association have suggested that people with poor sleep quality may be more likely to use food as a way to cope with negative emotions, which, in turn, may be collaterally associated with dietary restraint in response to overeating to prevent weight gain [37]. At the neurological level, deficient sleep can activate brain regions that are receptive to hedonic food stimuli, which may be related to increased motivation to seek food as a rewarding method [38]. Although inversely, our results of the association between higher sleep quality and less prevalence of increased CR and EE might be in line with that observed in previous research. However, we do not have an explanation for the association between lower sleep quality and less prevalence of CR and EE. The same intriguing association was observed for higher tobacco consumption. Higher consumption of tobacco was negatively associated with higher CR and EE. Although further investigation is required to explore this association in depth, we can speculate that COVID-19 confinement might have facilitated the increase in smoking to relieve emotional distress and anxiety instead of eating in response to stressful conditions. However, to our knowledge, this hypothesis remains untested.

### Limitations and Strengths

This study presents some limitations and strengths that should be considered. The first limitation is that the cross-sectional nature of our study does not permit the establishment of a cause–effect relationship from the results obtained. In addition, we did not collect information about the previous mental health problems as a proxy for increased CR, EE, or UE, which would allow us to exclude them from the analysis. Therefore, we should not disregard possible reverse causation. However, little is known about the factors associated with CR, EE, and UE; thus, this study can help promote programs to reduce undesirable eating behaviours in social isolation situations such as COVID-19 confinement. Moreover, in terms of research, this study can provide in parallel a rationale for potential replication in other samples using a longitudinal study design. Another limitation was that using a self-reported survey can cause recall bias and social desirability effects. In addition, we used a cross-cultural adaptation of the TFEQ-R18-SP conducted only with individuals from Spain and not from other Spanish-speaking countries, which could involve slight cultural differences in understanding. However, using this validated questionnaire of eating behaviours can help to minimise these potential biases; therefore, any inaccuracy in reporting should be non-differential. In our study, people with low educational levels and men were underrepresented. However, this situation should not affect the predictor factors associated with eating behaviours. We should also acknowledge that the sociodemographic and lifestyle variables were measured using ad hoc questions; therefore, the study findings should be interpreted with caution. The main strength of this study was the large sample size of Spanish-speaking populations in different countries. Moreover, the heterogeneity in the variables collected allowed us to classify people in various levels of exposure. However, the sample in our survey was a convenience sample with a high proportion of well-educated women and the recruitment method excluded the “offline” population that does not use the internet. Hence, the lack of representativeness prevents results from being able to apply to the entire Spanish-speaking population. Finally, the statistical and methodological procedures ensure the accuracy and validity of the results by providing convincing evidence.

## 5. Conclusions

In conclusion, this study offers information about eating behaviour during the first confinement pandemic of COVID-19 from 21 Spanish-speaking countries. In addition, the results may contribute to understanding the effect of confinement on problematic eating behaviours and help to design interventions to promote healthy lifestyles.

## Figures and Tables

**Table 1:** Sociodemographic and lifestyle characteristics of the participants in the CoV-Eat project.

Characteristics	*n* = 9849
Age in years, median (IR ^1^)	31 (27; 43)
Sex (women), %	80.1
Marital status (with partner), %	93.4
Educational level (≤primary), %	18.6
Household income, %	
Lower	17.1
Medium	75.6
Higher	7.3
Number of people in home (>2), %	66.5
Days of confinement, median (IR)	23 (17; 36)
Numbers time left their home in the last week, %	
0	15.2
1	33.4
>1	51.3
Sleep quality, %	
As before	46.4
Higher	11.7
Lower	41.9
Physical activity, %	
As before	21.5
Higher	16.2
Lower	62.3
Screen use, %	
As before	24.5
Higher	72.4
Lower	3.1
Tobacco consumption, %	
Non-smoker	64.9
As before	4.4
Higher	4.8
Lower	6.0
Ex-smoker	20.0
Relationship with food, %	
As before	47.6
Different	52.4

^1^ IR: Interquartile range.

**Table 2:** Associated factors with medium (15–16 points) and high (>16 points) scores of cognitive restrained in the participants in the CoV-Eat project (*n* = 9849).

		<15 Points (*n* = 3593)	15–16 Points (*n* = 3420)			>16 Points (*n* = 2836)		
		%	%	PR (95% CI)	*p*-Value	%	PR (95% CI)	*p*-Value
Age in years	18–30	33.6	37.7	1.00		39.9	1.00	
	31–40	32.2	30.7	0.92 (0.86; 0.97)	0.003	30.5	0.91 (0.85; 0.97)	0.006
	41–65	34.3	31.6	0.90 (0.84; 0.95)	0.001	29.6	0.83 (0.78; 0.89)	<0.001
Sex	Woman	83.8	79.9	1.00		75.5	1.00	
	Man	16.2	20.1	1.14 (1.07; 1.21)	<0.001	24.5	1.30 (1.22; 1.38)	<0.001
Marital status	Coupled up	92.7	93.9	1.00		93.8	1.00	
	Single	7.3	6.1	0.95 (0.85; 1.06)	0.357	6.2	1.01 (0.90; 1.14)	0.881
Education level	≤primary	18.5	17.3	1.00		20.1	1.00	
	>primary	81.5	82.7	1.05 (0.98; 1.12)	0.191	79.9	0.97 (0.91; 1.04)	0.371
Household income	Lower	16.5	16.0	1.00		19.0	1.00	
	Medium	75.5	76.5	1.00 (0.94; 1.08)	0.894	74.7	0.90 (0.84; 0.96)	0.002
	Higher	8.0	7.5	0.96 (0.86; 1.07)	0.458	6.3	0.80 (0.70; 0.91)	0.001
People living at home	0–2	33.1	34.5	1.00		32.8	1.00	
	>2	66.9	65.5	0.96 (0.91; 1.01)	0.128	67.2	0.98 (0.92; 1.04)	0.493
Days of confinement	7–21 days	33.2	34.8	1.00		37.1	1.00	
	22–31 days	31.8	33.1	0.98 (0.93; 1.04)	0.587	33.0	0.92 (0.86; 0.98)	0.010
	>31 days	35.0	32.1	0.96 (0.90; 1.02)	0.166	29.9	0.90 (0.84; 0.97)	0.003
Number of times leaving home during the last week	0	13.9	15.3	1.00		16.9	1.00	
	1	33.6	33.6	0.95 (0.88; 1.02)	0.177	33.0	0.91 (0.84; 0.98)	0.014
	>1	52.5	51.1	0.93 (0.86; 0.99)	0.032	50.1	0.87 (0.81; 0.94)	<0.001
Sleep quality	As before	41.7	46.1	1.00		52.7	1.00	
	Higher	12.4	11.4	0.92 (0.85; 1.00)	0.047	11.1	0.88 (0.80; 0.96)	0.004
	Lower	45.9	42.5	0.94 (0.90; 1.00)	0.035	36.2	0.87 (0.82; 0.92)	<0.001
Physical activity	As before	19.8	21.4	1.00		23.8	1.00	
	Higher	15.8	15.9	0.98 (0.90; 1.06)	0.534	16.9	1.01 (0.93; 1.09)	0.874
	Lower	64.3	62.7	0.99 (0.93; 1.05)	0.668	59.2	0.97 (0.90; 1.03)	0.283
Screen use	As before	22.4	23.2	1.00		28.6	1.00	
	Higher	74.8	73.6	1.00 (0.94; 1.06)	0.894	67.9	0.91 (0.85; 0.96)	0.001
	Lower	2.8	3.2	1.11 (0.97; 1.28)	0.125	3.5	1.08 (0.94; 1.24)	0.288
Tobacco consumption	No smoker	61.6	64.1	1.00		69.9	1.00	
	As before	4.5	4.5	0.99 (0.88; 1.11)	0.813	4.1	0.88 (0.77; 1.01)	0.075
	Higher	5.7	4.6	0.91 (0.80; 1.02)	0.114	3.8	0.81 (0.69; 0.94)	0.006
	Lower	6.3	6.0	0.97 (0.87; 1.07)	0.538	5.5	0.87 (0.77; 0.98)	0.020
	Ex-smoker	21.9	20.8	0.98 (0.92; 1.04)	0.560	16.6	0.83 (0.77; 0.89)	<0.001
Relationship with food	As before	39.9	46.1	1.00		59.1	1.00	
	Different	60.1	53.9	0.89 (0.85; 0.94)	<0.001	40.9	0.69 (0.65; 0.73)	<0.001

PR, Prevalence Ratio; CI, Confidence Interval.

**Table 3:** Associated factors with medium (9–10 points) and high (>10 points) scores of emotional eating in the participants in the CoV-Eat project (*n* = 9849).

		<9 Points (*n* = 4395)	9–10 Points (*n* = 2874)			>10 Points (*n* = 2580)		
		%	%	PR (95% CI)	*p*-Value	%	PR (95% CI)	*p*-Value
Age in years	18–30	38.9	37.9	1.00		32.0	1.00	
	31–40	32.7	29.2	0.95 (0.89; 1.02)	0.172	30.9	0.95 (0.90; 1.00)	0.032
	41–65	28.4	32.9	1.08 (1.01; 1.16)	0.024	37.1	0.92 (0.87; 0.97)	0.002
Sex	Women	85.0	79.1	1.00		72.8	1.00	
	Men	15.0	20.9	1.22 (1.14; 1.3)	<0.001	27.2	1.11 (1.06; 1.17)	<0.001
Marital status	Coupled up	93.7	93.6	1.00		92.6	1.00	
	Single	6.3	6.4	1.03 (0.92; 1.16)	0.613	7.4	0.97 (0.89; 1.07)	0.581
Education level	≤primary	19.3	16.9	1.00		19.2	1.00	
	>primary	80.7	83.1	1.09 (1.01; 1.18)	0.024	80.8	1.02 (0.96; 1.08)	0.508
Household income	Lower	18.8	15.3	1.00		16.1	1.00	
	Medium	73.9	76.9	1.09 (1.00; 1.18)	0.052	77.1	0.98 (0.92; 1.03)	0.428
	Higher	7.4	7.8	1.07 (0.95; 1.22)	0.268	6.8	0.94 (0.85; 1.04)	0.223
People living at home	0–2	33.1	33.9	1.00		33.9	1.00	
	>2	66.9	66.1	0.98 (0.92; 1.04)	0.518	66.1	0.99 (0.94; 1.03)	0.594
Days of confinement	7–21 days	33.2	35.5	1.00		37.1	1.00	
	22–31 days	30.9	33.2	0.97 (0.91; 1.04)	0.361	34.7	0.98 (0.93; 1.03)	0.391
	>31 days	35.9	31.3	0.93 (0.87; 1.00)	0.047	28.2	0.97 (0.92; 1.02)	0.202
Number of times leaving home during the last week	0	14.9	14.7	1.00		16.4	1.00	
	1	34.2	33.1	0.98 (0.90; 1.07)	0.651	32.6	0.95 (0.89; 1.01)	0.087
	>1	50.9	52.2	0.97 (0.89; 1.05)	0.472	51.0	0.92 (0.87; 0.97)	0.004
Sleep quality	As before	39.7	49.5	1.00		54.5	1.00	
	Higher	11.2	11.6	0.96 (0.88; 1.05)	0.38	12.6	0.92 (0.86; 0.99)	0.023
	Lower	49.2	38.9	0.88 (0.82; 0.93)	<0.001	32.9	0.95 (0.91; 1.00)	0.031
Physical activity	As before	17.3	23.6	1.00		26.4	1.00	
	Higher	13.8	17.2	1.02 (0.94; 1.11)	0.603	19.1	0.99 (0.92; 1.06)	0.743
	Lower	68.9	59.2	0.86 (0.8; 0.92)	<0.001	54.5	0.99 (0.94; 1.04)	0.747
Screen use	As before	19.0	27.0	1.00		31.0	1.00	
	Higher	78.5	70.3	0.85 (0.80; 0.91)	<0.001	64.4	0.97 (0.92; 1.02)	0.203
	Lower	2.5	2.7	0.99 (0.83; 1.17)	0.867	4.6	1.05 (0.94; 1.18)	0.375
Tobacco consumption	No smoker	62.3	66.1	1.00		67.8	1.00	
	As before	4.2	4.5	1.00 (0.87; 1.14)	0.969	4.7	0.97 (0.87; 1.08)	0.596
	Higher	5.3	5.0	1.00 (0.88; 1.14)	0.974	3.6	0.87 (0.78; 0.98)	0.026
	Lower	6.6	5.6	0.92 (0.81; 1.05)	0.222	5.4	0.94 (0.86; 1.03)	0.184
	Ex-smoker	21.7	18.8	0.88 (0.82; 0.95)	0.001	18.5	0.98 (0.93; 1.04)	0.587
Relationship with food	As before	32.9	53.1	1.00		66.5	1.00	
	Different	67.1	46.9	0.67 (0.63; 0.71)	<0.001	33.5	0.91 (0.87; 0.95)	<0.001

PR, Prevalence Ratio; CI, Confidence Interval.

**Table 4:** Associated factors with medium (24–26 points) and high (>26 points) scores of uncontrolled eating in the participants in the CoV-Eat project (*n* = 9849).

		<24 Points (*n* = 4086)	24–26 Points (*n* = 2508)			>26 Points (*n* = 3255)		
		%	%	PR (95% CI)	*p*-Value	%	PR (95% CI)	*p*-Value
Age in years	18–30	40.8	36.4			32.1		
	31–40	31.6	31.6	1.08 (1.00; 1.17)	0.040	30.3	0.96 (0.91; 1.01)	0.083
	41–65	27.6	31.9	1.18 (1.09; 1.28)	<0.001	37.5	0.93 (0.89; 0.98)	0.007
Sex	Women	80.3	79.6			80.2		
	Men	19.7	20.4	0.98 (0.91; 1.06)	0.573	19.8	1.11 (1.06; 1.16)	<0.001
Marital status	Coupled up	94.1	93.6			92.4		
	Single	5.9	6.4	1.03 (0.91; 1.17)	0.634	7.6	0.97 (0.89; 1.06)	0.565
Education level	≤primary	20.8	16.7			17.2		
	>primary	79.2	83.3	1.16 (1.06; 1.26)	0.001	82.8	1.01 (0.96; 1.06)	0.757
Household income	Lower	19.0	15.5			15.8		
	Medium	74.3	76.6	1.06 (0.97; 1.16)	0.195	76.4	1.00 (0.95; 1.06)	0.897
	Higher	6.7	7.9	1.14 (1.00; 1.31)	0.051	7.7	0.98 (0.89; 1.07)	0.658
People living at home	0–2	32.3	33.7			34.9		
	>2	67.7	66.3	0.97 (0.91; 1.03)	0.329	65.1	0.98 (0.94; 1.02)	0.299
Days of confinement	7–21 days	31.6	35.2			38.7		
	22–31 days	32.1	34.3	0.95 (0.88; 1.02)	0.149	32.0	0.97 (0.93; 1.02)	0.269
	>31 days	36.3	30.5	0.87 (0.81; 0.94)	<0.001	29.3	0.95 (0.91; 1.00)	0.064
Number of times leaving home during the last week	0	14.8	15.7	1.00		15.4	1.00	
	1	33.9	32.5	0.94 (0.85; 1.03)	0.159	33.6	0.96 (0.91; 1.02)	0.222
	>1	51.2	51.8	0.93 (0.85; 1.02)	0.131	51.0	0.94 (0.89; 1.00)	0.033
Sleep quality	As before	40.5	47.2	1.00		53.2	1.00	
	Higher	10.8	13.0	1.07 (0.97; 1.17)	0.157	11.8	0.96 (0.90; 1.02)	0.206
	Lower	48.7	39.7	0.90 (0.84; 0.96)	0.002	35.1	0.95 (0.91; 0.99)	0.029
Physical activity	As before	17.9	23.2	1.00		24.9	1.00	
	Higher	13.3	17.4	1.06 (0.96; 1.16)	0.249	18.8	1.01 (0.94; 1.07)	0.844
	Lower	68.8	59.4	0.86 (0.80; 0.93)	<0.001	56.3	1.00 (0.95; 1.05)	0.911
Screen use	As before	19.0	25.3	1.00		30.7	1.00	
	Higher	78.7	71.5	0.86 (0.81; 0.92)	<0.001	65.1	0.99 (0.94; 1.04)	0.634
	Lower	2.3	3.2	1.11 (0.93; 1.32)	0.237	4.1	1.09 (0.98; 1.21)	0.124
Tobacco consumption	No smoker	62.2	64.7	1.00		68.4	1.00	
	As before	4.3	4.3	0.97 (0.84; 1.12)	0.682	4.5	0.98 (0.89; 1.08)	0.723
	Higher	5.7	4.5	0.90 (0.77; 1.05)	0.186	3.7	0.93 (0.84; 1.03)	0.169
	Lower	6.9	5.8	0.93 (0.81; 1.07)	0.306	5.0	0.95 (0.86; 1.04)	0.226
	Ex-smoker	20.9	20.7	0.95 (0.88; 1.03)	0.198	18.4	0.98 (0.93; 1.03)	0.396
Relationship with food	As before	34.1	49.3	1.00		63.2	1.00	
	Different	65.9	50.7	0.73 (0.69; 0.78)	<0.001	36.8	0.90 (0.86; 0.94)	<0.001

PR, Prevalence Ratio; CI, Confidence Interval.

## Data Availability

Data will be made available on request.
